# Measuring affect-related cognitive bias: Do mice in opposite affective states react differently to negative and positive stimuli?

**DOI:** 10.1371/journal.pone.0226438

**Published:** 2019-12-30

**Authors:** Anna C. Trevarthen, Sarah Kappel, Claire Roberts, Emily M. Finnegan, Elizabeth S. Paul, Isaac Planas-Sitjà, Michael T. Mendl, Carole Fureix

**Affiliations:** 1 Bristol Veterinary School, University of Bristol, Bristol, United Kingdom; 2 School of Biological & Marine Science, University of Plymouth, Plymouth, Devon, United Kingdom; 3 Department of Biological Sciences, Graduate School of Science and Engineering, Tokyo Metropolitan University, Tokyo, Japan; University of Portsmouth, UNITED KINGDOM

## Abstract

Affect-driven cognitive biases can be used as an indicator of affective (emotional) state. Since humans in negative affective states demonstrate greater responses to negatively-valenced stimuli, we investigated putative affect-related bias in mice by monitoring their response to unexpected, task-irrelevant stimuli of different valence. Thirty-one C57BL/6J and 31 DBA/2J females were individually trained to return to their home-cage in a runway. Mice then underwent an affective manipulation acutely inducing a negative (NegAff) or a comparatively less negative (CompLessNeg) affective state before immediately being tested in the runway with either an ‘attractive’ (familiar food) or ‘threatening’ (flashing light) stimulus. Mice were subsequently trained and tested again (same affective manipulation) with the alternative stimulus. As predicted, mice were slower to approach the light and spent more time with the food. DBA/2J mice were slower than C57BL/6J overall. Contrary to predictions, NegAff mice tended to approach both stimuli more readily than CompLessNeg mice, especially the light, and even more so for DBA/2Js. Although the stimuli successfully differentiated the response of mice to unexpected, task-irrelevant stimuli, further refinement may be required to disentangle the effects of affect manipulation and arousal on the response to valenced stimuli. The results also highlight the significant importance of considering strain differences when developing cognitive tasks.

## Introduction

A key goal in animal welfare science is to measure the affective (emotional) states of animals since these are a major determinant of an individual’s well-being. Affective states are valenced (*i*.*e*. they are positive or negative) and they comprise behavioural, neural, physiological and subjective components [1]. The subjective ‘feelings’ component can be assessed *via* linguistic report in humans, but this is not possible in non-human animals. Instead, we must assess animal affect only in terms of its other components, and infer (or not infer) the subjective component according to other considerations (*e*.*g*. see [2]). In humans, self-reported affect is associated with variation in cognitive processes in the form of judgement, memory and attention biases. For example, people reporting negative affect make more negative judgements about ambiguous events (*e*.*g*. [3,4]), pay more attention to threatening stimuli (*e*.*g*. [5–8]), and recall more negative memories (*e*.*g*. [9,10]) than those reporting more positive affect (see [1] for review). There is evidence for a similar relationship between affective valence (positivity vs negativity) and judgement under ambiguity in animals ([11–15]); such ‘judgement biases’ can thus be useful behavioural indicators of affective valence in animals.

Since the seminal work of Harding et al. (2004), methods for assessing cognitive judgment biases in animals have been developed for a diverse range of species including rodents (see reviews [16] and [17]), primates (*e*.*g*. [18,19]), birds (*e*.*g*. [20,21]) and invertebrates (*e*.*g*. [22–27]). However, most of these methods require lengthy periods of discrimination training, in which animals are taught to perform operant tasks to respectively access and avoid positively- and negatively-valenced stimuli (*e*.*g*. Go/No-Go tasks). This can make such protocols impractical for assessing the affective states of large numbers of animals [28]. Additionally, a significant proportion of individuals can fail to achieve rigorous discrimination in such tasks [29], and negative experiences during training can influence the response to subsequent ambiguous cues during testing [30]. Efforts have been made to refine protocols for measuring cognitive judgement bias in animals, including by reducing the length of habituation and discrimination training phases, using tasks involving positive rewards of differing value (and avoiding negative stimuli) (*e*.*g*. [31–33]), and designing tasks that take advantage of naturalistic stimuli or behaviour (*e*.*g*. [30,34,35]). Nevertheless, many judgement bias tasks remain lengthy to train (e.g. on average 27 trials [14], see also [33]) and implement.

Simpler and potentially quicker protocols in which individuals show enhanced or reduced attention to particular stimuli according to affective state, show promise. Evidence of affect-related attention biases have been suggested for both humans (*e*.*g*. see reviews [36–38]) and other species (starlings: [39,40]; parrots: [41]; sheep: [28,42]; cattle: [43]; macaques: [44]). Although there has been a lack of agreement about the cognitive mechanisms underlying these biases in humans [3,37,45,46], it is widely agreed that high levels of anxiety and other negative affective disorders are linked with a greater response (*e*.*g*. attention [36,45,47,48] and distractibility [6,49,50]), specifically to task-irrelevant *threatening* stimuli. In contrast, people with more optimistic reward expectancies (associated with positive affect [51]) show greater attentional bias towards *rewarding* than punishing stimuli [52–54].

Measuring attention in humans often involves establishing how and when a stimulus is perceived using linguistic report or by using behavioural or physiological indicators, such as eye gaze or reaction time. Accurately identifying attention-related changes in such processes becomes more difficult for non-human species, whose sensory and perceptual systems may differ greatly from our own and can be difficult to interpret [55]. Using mice as a model species, we therefore aimed to develop a simple and novel protocol for assessing affect-related biases in their response to unexpected task-irrelevant stimuli that were designed to be either mildly threatening (a bright flashing light) or mildly rewarding (a familiar food item).

Developing new, non-invasive tools for inferring affective state is of importance to animal welfare scientists, neuroscientists and psychopharmacologists interested in the assessment of animal affect. Validated measures of affective state can help to develop improved models and treatment options for human emotional disorders [38,56], as well as providing additional information regarding the structure and functions of animal affect, against which other measures of animal welfare can be compared. Mice represent the ideal species for the development of such measures as they are the primary mammalian model for neuropsychological research [57] and conducting an acute affective manipulation on this species in a controlled environment is relatively straightforward.

Our objective in the present study was to develop a novel measure of cognitive bias in mice by assessing their response to unexpected, task irrelevant stimuli (i.e. distractors) of differing valence. We aimed to establish a protocol that would require minimal training and would be transferrable to different strains of mice, and (following validation) to different species. We therefore worked with two commonly used strains of laboratory mouse: C57BL/6J and DBA/2J [58], which differ in their behaviour and apparent anxiety levels (with some differences between studies: *e*.*g*. [59–64]) and their physical appearance (aiding identification in mixed-strain housing refinement) and. Because females are at a significantly greater risk of developing affect-related disorders in humans [65], we used female mice for this study (additionally, females of both strains are known to be able to be mixed-housed with no detrimental impact on welfare [66]).

We developed a runway task in which mice were trained to return to their home cage (positive reinforcer for mice [56,67]). Mice then underwent either a negative [68] or a comparatively less negative affect manipulation [69], immediately followed by a runway test in which a mildly ‘threatening’ (flashing white light, which is preferentially avoided by mice) [70–72] or a mildly ‘attractive’ (familiar food) [73] stimulus was unexpectedly added. Mice were subsequently subjected to a second test with the same affect manipulation as previously, but with the opposite stimulus type. Given the putative difference between the two unexpected stimuli, we hypothesized that mice would take longer to approach the flashing light than the food, and spend more time in proximity to the familiar food than the flashing light. Drawing our predictions from the phenomenon in humans, we expected that mice in a negative affective state would be more attentive to (i.e. more distracted by) the ‘threatening’ light stimulus than mice exposed to a comparatively less negative affective state manipulation. We thus predicted that mice in a negative affective state would take longer to approach the light and to reach the home-cage than those exposed to a comparatively less negative affective state manipulation. Additionally, we predicted that mice exposed to a comparatively less negative affective state manipulation would take less time to approach and spend more time consuming food than mice in a negative affective state.

## Materials and methods

### Ethical statement

This study was conducted at the University of Bristol under UK Home Office Licence (PPL: P2556FBFE). The Home Office application and all protocols used were given approval by the University of Bristol Animal Welfare and Ethical Review Body. Animal use and care was in accordance with the Animals (Scientific Procedures) Act 1986, EU directive 2010/63/EU and UK Home Office code of practice for the housing and care of animals bred, supplied or used for scientific purposes. All animals were weighed weekly and monitored daily throughout the study for any health issues. At the end of the study (after 14 weeks), the animals were euthanised by skilled technicians using a schedule 1 method, (concussion, immediately followed by cervical dislocation and confirmation of death–the use of both methods ensured rapid loss of consciousness).

### Animals, housing and husbandry

Thirty-one female C57BL/6J and 31 female DBA/2J mice (*Mus musculus*; Charles River, France) were housed in 31 highly enriched transparent cages (*Techniplast*, dimensions: 44cm L x 34cm W x 20cm H) from three weeks of age (sample sizes based on power analyses using data previously published in [66]). Upon arrival in the laboratory, each mouse was weighed and one C57BL/6J (black) and one DBA/2J (brown) mouse of similar weight (+/- 2.5g) was pseudo-randomly (mouse selected at random, but matched with cage mate based on weight) allocated to each cage, to avoid the possibility of cage effects confounding strain effects. In addition, the mixed-strain housing enabled individual identification within pairs, removing the need for invasive marking procedures. The cages contained sawdust (*IPS*), nesting material, a cardboard shelter (13cm L x 13cm W x 13cm H, *Little Cherry Ltd*), a red igloo with a running wheel (fast-trac, *Datesand*), two cardboard tubes (7.5cm L, ∅ 3.8cm, *IPS*), a transparent polycarbonate handling tunnel (13cm L, ∅ 5cm, *Datesand*), a flexible plastic tube (approximately 20cm long, ∅ 7.5cm, *SnuggleSafe*) which was attached to the cage lid, three aspen gnawing blocks (two small: 5cm L x 1cm W x 1cm H, one large: 10cm L x 2cm W x 2cm H, *Datesand*), two nestlets (*Ancare*, USA), a hammock (roughly 12 cm x 12 cm) made from a sock attached to the lid, a sisal rope ladder and one half of a coconut shell (approximate dimensions: 12.7cm L x 7.6cm W x 5cm H, *Little Cherry Ltd*) which was attached to the lid with sisal rope. Other natural materials (two small pine cones, two wooden sticks (10cm L)) were sterilised in an autoclave before being added to each cage for additional enrichment. A millet spray and one cob of dried corn (*Pets at Home*) was attached to the lid of each cage. Food treats (*e*.*g*. oatmeal crisps with maple syrup, *Pets at Home*) were given on a daily basis in the home cage. Food (*LabDiet*) and water were available *ad-libitum* and animals were kept under a 12hr reversed light-dark cycle (lights on 1900–0700). All cages were housed within three Scantainers (*Scanbur BK*) in the home room and were cleaned every four weeks. Temperature (Mean ± SEM, 20.9± 0.5°C) and relative humidity were controlled at the room level (Scantainers used as shelving units with doors open) and were checked daily.

### Handling habituation

During the first four weeks, mice were gradually habituated to both the presence of the experimenters (5 sessions) and to tunnel handling [68,69,74] (13 sessions). During handling, experimenters always wore gloves and a white laboratory coat. Each cage was removed from the Scantainer and placed on a trolley in the centre of the home room, where handling took place. At all times, mice were handled using the polycarbonate tunnel from their home cage, following a validated method shown to reduce stress in laboratory mice [68,69,74]. To initially encourage mice into the tunnel, a few droplets of condensed milk (Carnation, *Nestle*) were dropped in the centre of the tunnel and smeared in a semicircle to allow both mice simultaneous access. One familiar food treat was given to each mouse following habituation / handling sessions. Each individual mouse’s progress was assessed and recorded on a daily basis (monitoring behaviour during habituation and handling until they reached the criteria: “confidently entered handling tunnel without encouragement and lifted out of the cage”). All handling and subsequent testing took place during the active (dark) phase (between 0900–1800), under red light.

### Test apparatus and training

**Fig 1:**
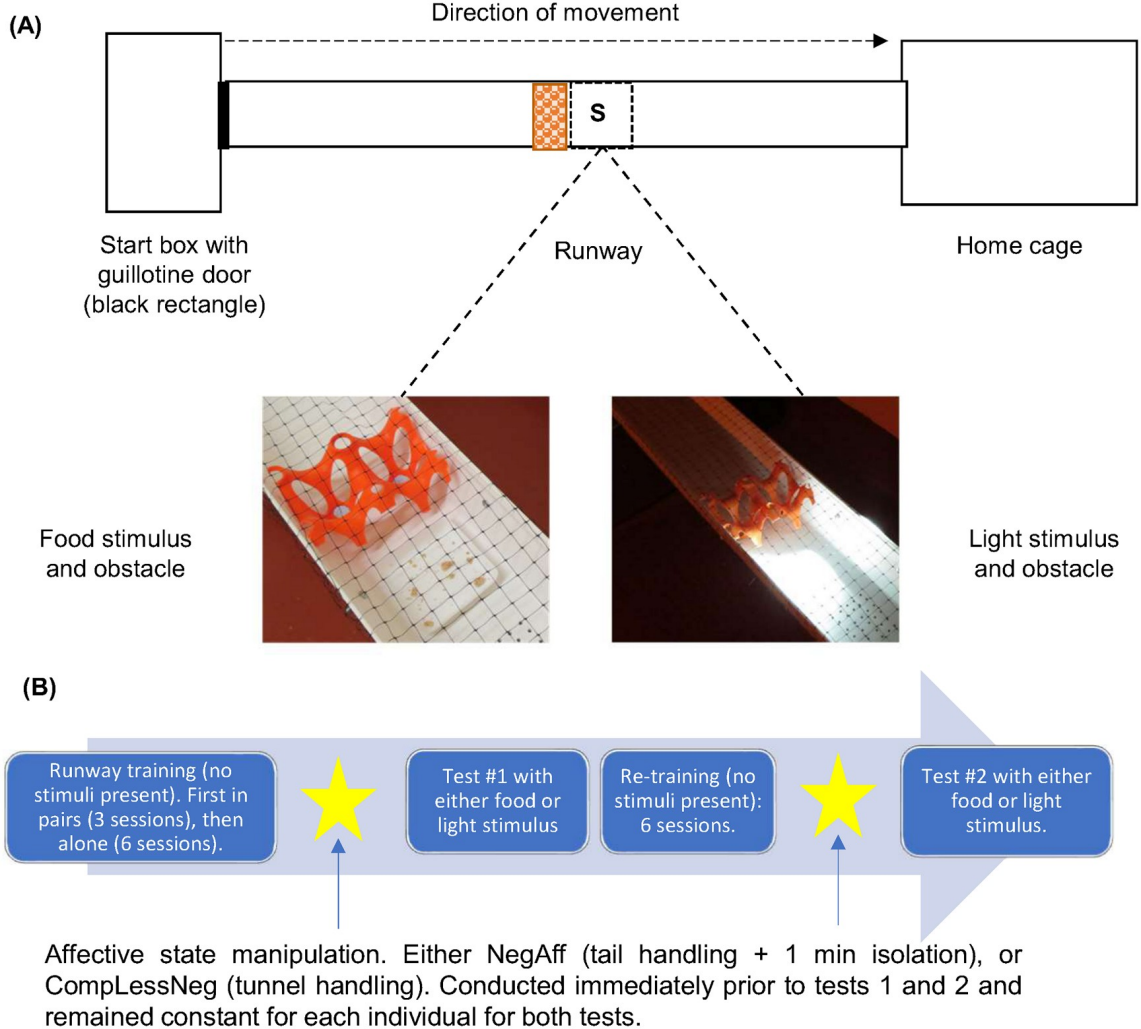
Runway set-up for test and experimental procedure. (A) Shows obstacle (orange egg carton) location and stimulus presentation area (location S) which was positioned at 118cm from the start-box door. Mice were exposed to either a familiar food or flashing light stimulus. All tests were conducted under red ambient light. (B) Experimental procedure and timeline.

The test apparatus consisted of a long white plastic runway (200cm L x 7cm W x 5.5cm H) which connected a start-box (33cm L x 15cm W x 10cm H) to the home cage (see Fig 1a) and was located in a testing room, which was isolated from the home room. A guillotine door separated the start-box from the rest of the runway. The runway was covered with a fine plastic mesh to ensure that the mice could not escape. They were first habituated to the test apparatus in pairs (*i*.*e*. cage mates) over three daily sessions. During paired habituation, both mice were moved (using the tunnel) into the start-box and the guillotine door was raised (approximately 10s later) and remained open to allow exploration of the apparatus. In the first two sessions the lid of the home cage was removed to allow the mice to re-enter it directly, but in the third session the home cage lid was closed and mice were transferred back to their home cage using the tunnel once they had made their way back to the home-cage end of the runway. The session ended when both mice had returned to the home cage or after 10 minutes had elapsed. Between sessions, the apparatus was cleaned with 70% ethanol.

After these three paired habituation daily sessions, runway training continued individually (training phase). During training, the mouse’s cage-mate was contained within the home cage at the end of the runway. On reaching the lid of the home cage, the mouse was caught in the handling tunnel and transferred back to the home cage. Each individual was given six training sessions (one per day). Latency to reach the home cage was recorded live for every trial. Cages of mouse pairs were tested in a pseudo-random order (using six predefined sequences to ensure each vertical Scantainer column and each shelf within each Scantainer were alternatively balanced), and the order that the cage mates were tested in was alternated across sessions.

### Testing and affective manipulation

The experimental timeline is outlined in Fig 1b.

On the day following the last training session, and immediately prior to the first test session, each mouse underwent an affective manipulation (either Negative: NegAff (N = 15 cages) or comparatively less negative: CompLessNeg (N = 16 cages)), which was pseudo-randomly allocated by an independent, naïve experimenter to each cage of mice (equally spreading NegAff and CompLessNeg affective state manipulation treatments within each vertical Scantainer column and within each shelf within each Scantainer). Cage mates were tested consecutively with the same affective manipulation (either NegAff or CompLessNeg), and the order cage mates were tested was alternated across tests. The NegAff manipulation involved an unfamiliar experimenter (CR, ‘negative’ handler) removing the test mouse from the home cage by picking it up at the base of the tail, which has been shown to be more aversive than handling using the tunnel [68] and isolating it for 1 minute in a small unfamiliar, barren gravel-bedded (preferentially avoided substrate, Fureix unpublished data) transparent box (15cm L x 10.5cm W x 10.5cm H; gravel: premium aquarium gravel, *Pets at Home*). Animals in the CompLessNeg condition were removed from the home cage using the tunnel following the same procedure as in habituation and training sessions by a familiar experimenter (AT, ‘less negative’ handler). Following these affective manipulations, each mouse was placed by its respective handler into the start-box of the runway apparatus to begin the test. The handler then left the room and remained blind to the stimulus used in the subsequent test. It was not possible to blind the test observer to the stimulus used in each test, but we subsequently conducted a reliability assessment on a subset of the latency data using a second naïve observer (Intraclass correlation coefficient = 0.998, df = 61, p<0.001).

By the end of the training phase, most mice quickly traversed the runway (median runway time for sixth training session: 9s, range: 5-24s). During testing, an unfamiliar obstacle was placed within the runway (a piece of a plastic egg tray 8.5cm L x 10cm W x 5cm H, 110cm away from the start-box–see Fig 1a), which served as a location marker for the unexpected stimuli (familiar food, N = 32 mice; flashing light, N = 30 mice), but also slowed mouse runway speed to ensure mice paid attention to the stimuli, which were situated at 118cm along the runway. The food was familiar condensed milk and a crumbled biscuit treat contained within a 6.5 x 6.5cm familiar plastic tray, and the light was a flashing white light beam (*EverBrite*), which has been previously used as a mild stress inducer for mice [70]. The stimulus type allocated to each mouse pair during this first test was pseudo-randomly pre-determined to ensure that the stimuli type was equally spread across shelves and columns within each Scantainer–i.e. to ensure all mice receiving food first were not clustered within a particular Scantainer location assigned by a blind experimenter. At the start of the test, the guillotine door of the start-box was raised as in training sessions, allowing the mouse to run to the home cage. Once the mouse had reached the end of the runway, the ‘less negative’ handler entered the room and carefully returned the mouse to the home cage using the handling tunnel. The test apparatus and obstacle were cleaned with 70% Ethanol after each test.

On the day following testing, mice began training in the runway apparatus again with no stimuli or obstacle present and this continued for six further sessions. A second test was then conducted, exposing each mouse to the alternative stimulus (*i*.*e*. light if first exposed to food or *vice-versa*), but combined with the same affective manipulation it was exposed to previously (see Fig 1b).

### Runway latency measures

Each mouse’s latency to return to the home cage from the time the guillotine door of the start box was fully raised was extracted for all trials (Fig 1a). To control for inter-individual differences in running speed, we calculated for each mouse its corrected runway latency for each test, i.e. the ratio of its test latency to its median latency during the six previous trials with no stimulus (either training for test 1 or re-training for test 2). For test sessions only, we also calculated the latency to approach the obstacle (adjacent to the stimulus presentation area–but removing the confound of time taken to cross the obstacle) and the time spent within the stimulus presentation area (S: Fig 1a).

All habituation, training, re-training and test sessions were video recorded using CCTV cameras (*Swann*; *Quadrox* Software). To ensure observer blinding, a tracking software tool (*USE Tracker*– www.usetracker.org) was used on test trials to extract the time spent in the stimulus presentation area. We planned to also use the tracking software to extract the latency to approach the obstacle and return to the home cage. However, visual inspections revealed that the obstacle and the home-cage goal line occasionally interfered with mouse detection in these zones, and we therefore relied on the manually-collected latencies for these two measures (Reliability Intraclass correlation coefficient = 0.998, df = 61, p<0.001).

On two occasions during training sessions (out of a total of 744 training and re-training trials), one mouse attempted to escape through the netting above the runway and the trial had to be stopped. Data collected during these trials were excluded from the following analyses. On four occasions (out of 124) during tests, the mouse failed to reach the home cage within the allotted 10min. For these trials a maximum latency of 600s was assigned.

### Statistical analysis

Data were analysed using R version 3.3.2 (R Core Team, 2016). Models were fitted using the package lme4 [75]. We constructed generalized linear mixed models with random intercepts, but fixed slopes. Within each model, cage and mouse were included as random effects (mouse nested within cage). Unexpected stimulus type (*i*.*e*. food or light), order of stimulus presentation (*i*.*e*. being tested first with food or light), affect manipulation (tunnel handling or tail handling plus isolation), strain (C57BL/6J or DBA/2J), stimulus*affect interaction, and the 3-way stimulus*affect*strain interaction were included as fixed effects. For each model, the assumptions of parametric testing were checked using Shapiro-Wilk normality tests on the residuals. Due to the exaggerated right-skew of the data, gamma distribution, rather than normal distribution models best represented the data and are presented throughout the Results section. Likelihood ratio tests were used to compare the full model, with models excluding each fixed term of interest. This allowed us to determine the significance of the term by measuring the deviance from the full model, using the Χ^2^ distribution.

## Results

**Fig 2:**
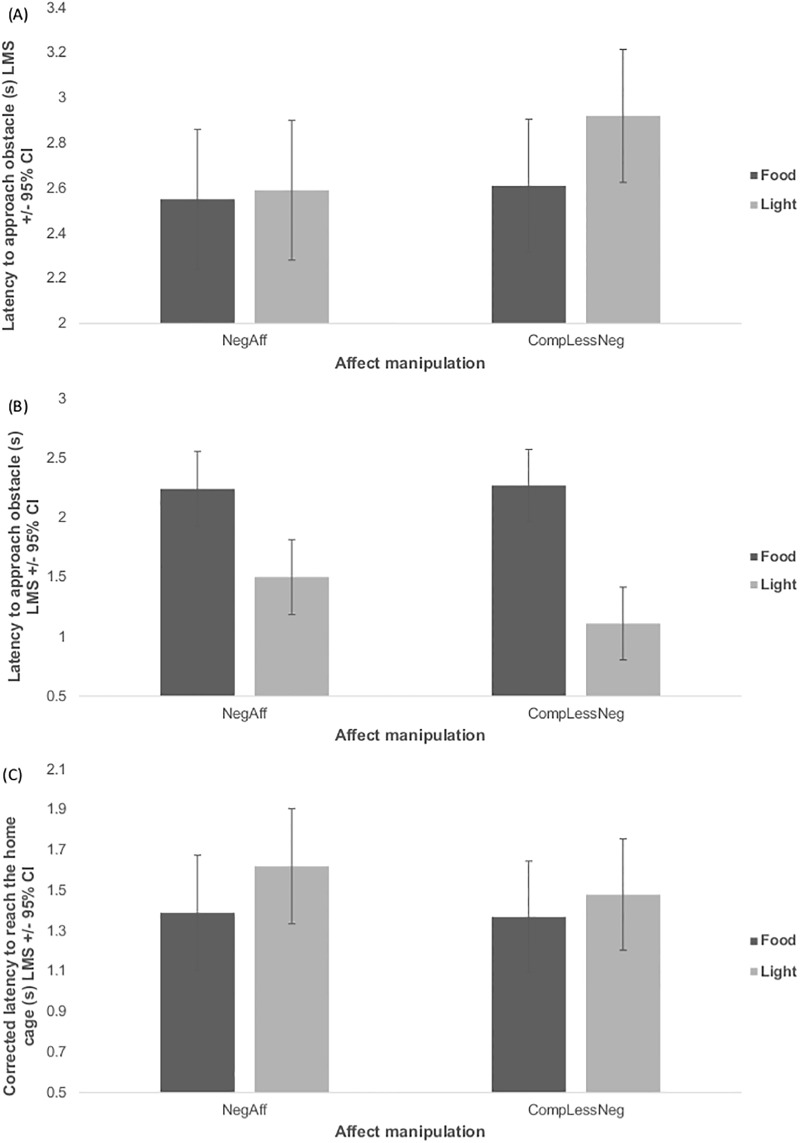
Least mean squares (s) +/- 95% confidence interval during tests split by stimulus type (either light or food). (A) Latency to approach the obstacle (adjacent to the stimulus presentation area), (B) time spent within the stimulus presentation area and (C) corrected latency to reach the home cage ‘Corrected latency’ is the ratio for each mouse of its test latency to its median latency during the six prior trials with no stimulus (either training for test 1 or re-training for test 2). Mice took longer to approach the obstacle with the light than the food stimulus (Χ^2^_(5)_ = 13.41, p = 0.020) and spent longer in the presence of the food than the light (Χ^2^_(5)_ = 31.49, p<0.001), but latency to reach the home cage did not significantly differ between stimuli (Χ^2^_(5)_ = 3.86, p = 0.570).

As predicted, there were significant main effects of stimulus type: mice took longer to leave the start box and approach the obstacle/stimulus when the light was present than when food was presented (Χ^2^_(5)_ = 13.41, p = 0.020) (Fig 2), and spent longer in the presence of the food than the light (Χ^2^_(5)_ = 31.49, p<0.001) (Fig 2). Perhaps due to these opposing effects, stimulus type had no significant effect on the corrected latency to reach the home cage (Χ^2^_(5)_ = 3.86, p = 0.570) (Fig 2).

**Fig 3:**
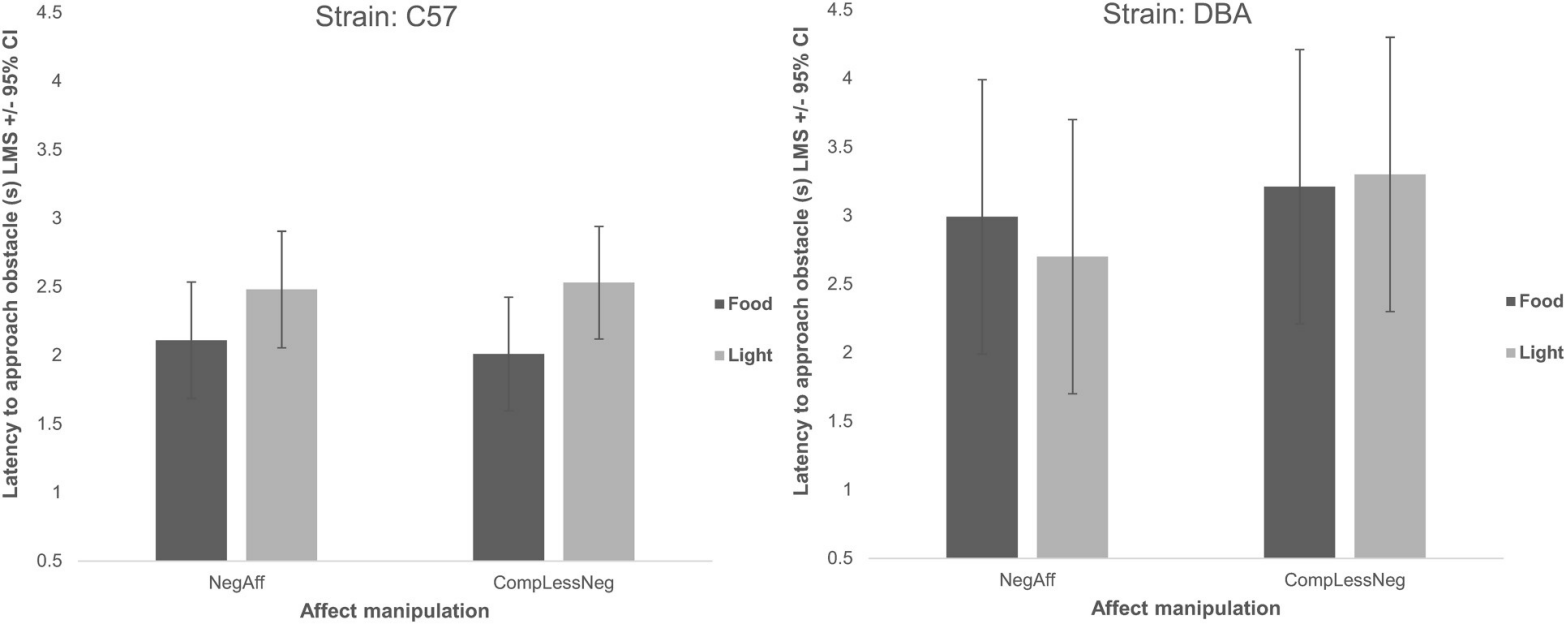
Latency to approach the obstacle split by strain (C57: C57BL/6J or DBA: DBA/2J), affect manipulation (negative: NegAff or comparatively less negative CompLessNeg), and stimulus type (either light or food) (s) (Least Mean Squares) +/- 95% Confidence Interval. DBA/2J mice took longer to approach overall than C57BL/6J mice (Χ^2^_(4)_ = 19.79, p<0.001), and latencies to approach tended to be shorter following NegAff manipulations (Χ^2^_(5)_ = 10.36, p = 0.066), especially with the light stimulus and even more so in DBA/2J mice (Χ^2^_(4)_ = 9.50, p = 0.050).

**Fig 4:**
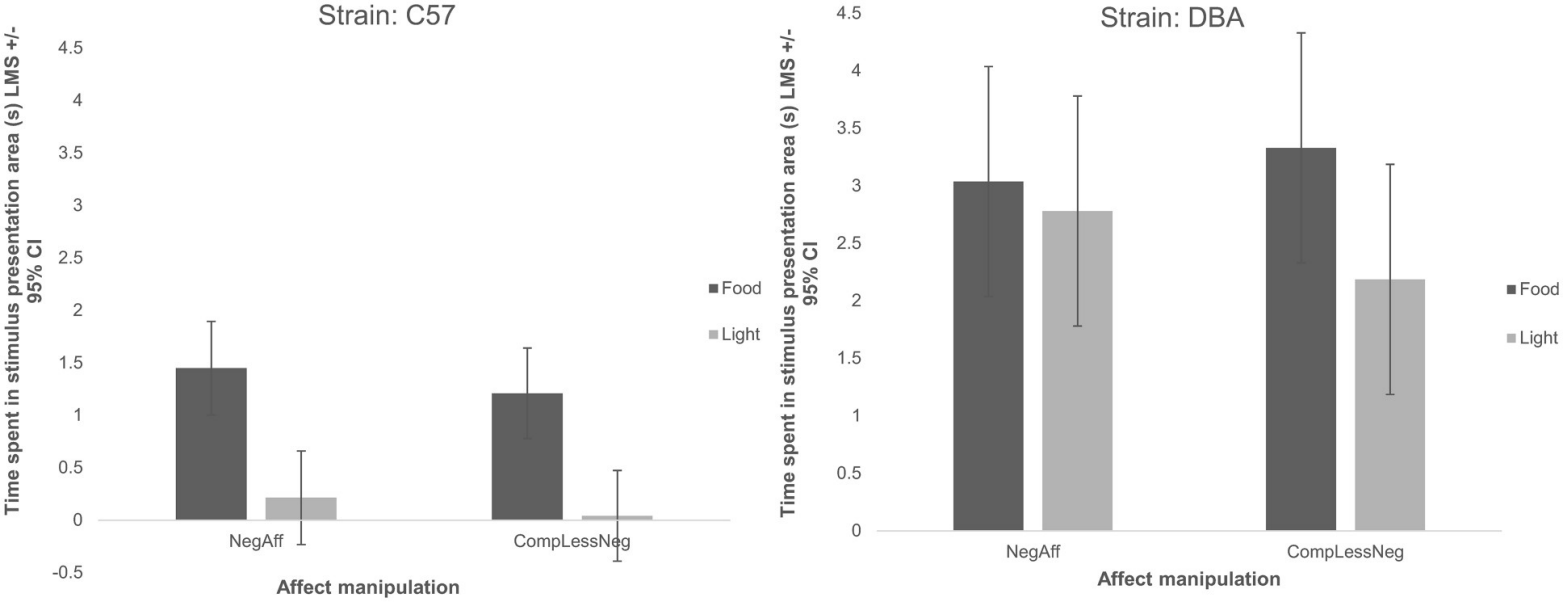
Time spent within the stimulus presentation area split by strain (C57: C57BL/6J or DBA: DBA/2J), affect manipulation (negative: NegAff or comparatively less negative CompLessNeg), and stimulus type (either light or food) (s) (Least Mean Squares) +/- 95% Confidence Interval. Mice spent longer in the stimulus presentation area with the food compared to the light (Χ^2^_(5)_ = 31.49, p<0.001), and DBA/2J mice spent longer within the stimulus presentation area than C57BL/6J mice (Χ^2^_(4)_ = 66.21, p<0.001); no other significant effect was observed.

**Fig 5:**
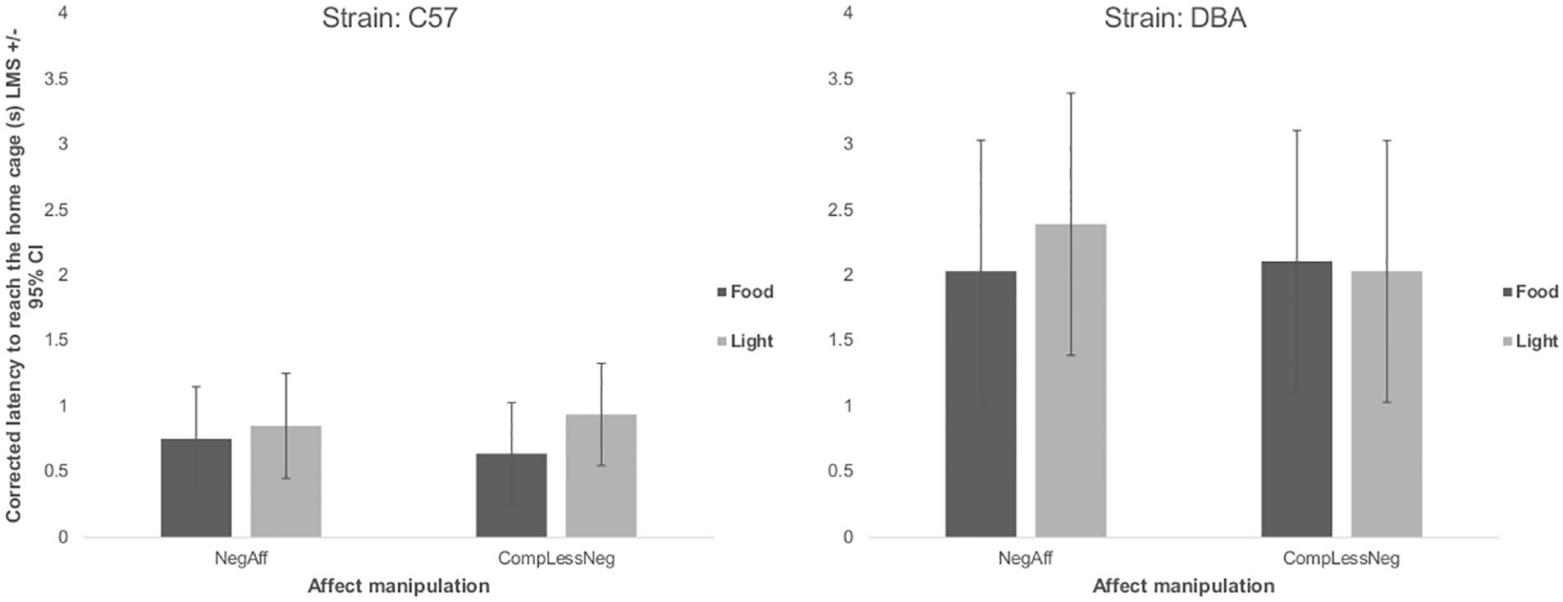
Corrected latency to reach the home cage split by strain (C57: C57BL/6J or DBA: DBA/2J), affect manipulation (negative: NegAff or comparatively less negative: CompLessNeg), and stimulus type (either light or food) (s) (Least Mean Squares) +/- 95% Confidence Interval. ‘Corrected latency’ is the ratio for each mouse of its test latency to its median latency during the six prior trials with no stimulus (either training for test 1 or re-training for test 2). DBA/2J mice had longer corrected latency to reach the home cage than C57BL/6J mice (Χ^2^_(4)_ = 31.44, p<0.001); no other significant effect was observed.

We also found, as expected, a significant main effect of strain: DBA/2J mice were slower to approach the obstacle, regardless of which stimulus was present (Χ^2^_(4)_ = 19.79, p<0.001) (Fig 3), spent longer in the presentation area (Χ^2^_(4)_ = 66.21, p<0.001) (Fig 4) and were slower to reach the home cage (Χ^2^_(4)_ = 31.44, p<0.001) than C57BL/6J mice (Fig 5).

Latencies to approach the obstacle tended to be shorter (though not statistically significantly so) following NegAff manipulations (Χ^2^_(5)_ = 10.36, p = 0.066) especially with the light stimulus (2-way interaction, Χ^2^_(4)_ = 9.50, p = 0.050), particularly for DBA/2J mice (3-way interaction, Χ^2^_(3)_ = 8.50, p = 0.037) (Fig 3). No significant main or interaction effect of affect manipulation was observed on the time spent in the presence of the two stimulus types (affect manipulation main: Χ^2^_(5)_ = 6.66, p = 0.245; affect*stimulus: Χ^2^_(4)_ = 5.59, p = 0.232; affect*stimulus*strain interaction: Χ^2^_(3)_ = 4.11, p = 0.249) (Fig 4). Moreover, contrary to the prediction that mice in a relatively negative affective state would be more sensitive to the light stimulus than mice exposed to a comparatively less negative affective state manipulation, we found no significant effect of affect manipulation (Χ^2^_(5)_ = 2.10, p = 0.835), affect*stimulus type (Χ^2^_(4)_ = 2.10, p = 0.717) or strain*affect*stimulus type (Χ^2^_(3)_ = 2.06, p = 0.560) on the corrected latency to reach the home cage (Fig 5).

## Discussion

We aimed to develop a novel measure of cognitive-bias by determining the response of mice (whose affective state was experimentally manipulated) to unexpected task-irrelevant stimuli. We expected mice to take longer to approach the light stimulus than the food and to spend more time in the proximity of the food than the light (this, we hypothesized, would be due to an avoidance reaction induced by the aversive nature of the light stimulus, and an approach reaction due to the positive nature of the familiar food). Additionally, we predicted that induced differences in the affective states of mice would generate affect-related cognitive biases and thus modulate their responses to the stimuli. We therefore predicted that mice in a negative affective state (NegAff) would be more sensitive to the light (take longer to approach and to reach the home-cage) than those exposed to a comparatively less negative affective state manipulation (CompLessNeg). Furthermore, we expected NegAff mice to take more time to approach and spend less time consuming food than CompLessNeg mice. Our predictions were partially supported, with mice taking longer to approach the light and spending less time with it, but we found no significant influence of affective manipulation on their response to the stimuli.

The runway task successfully induced differential effects in response to the stimuli. As predicted, mice were slower to approach the obstacle when the light was present than when food was present, and were less likely to remain in the stimulus presentation area (*i*.*e*. transitioning through this area more quickly) when the light was present compared to when the food was present. Stimulus type however had no significant effect on the overall runway latency. It is likely that the runway latency was not a sensitive enough measure to detect differences because the opposing effects on latency at different areas of the runway counteracted each other (for both stimuli), resulting in no observed difference in overall transit time. A task refinement could be to present both stimuli simultaneously to identify which stimulus the mouse approaches and investigates more readily. Additionally, the light stimulus may have presented more obvious visible cues than the food when mice were in the start-box, although we expected that the food would provide an olfactory cue. This could account for the slower approach time to the light, but not the lack of difference in overall runway latency. Future work could aim to use stimuli which provide equally salient visual and olfactory cues.

Although the two stimuli used in the task were successful in producing the predicted responses, we are less certain about the efficacy of the affective manipulations used. The only near-significant influence of affect manipulation (and significant interaction) was that mice (particularly DBA/2Js), were slower to approach both stimuli (particularly the light) following the comparatively less negative affective state manipulation. This might be in line with the report that humans in a comparatively positive affective state can demonstrate non-specific attention broadening towards task irrelevant information [76,77], perhaps because the environment is perceived as safe to explore [77], but does not match with greater attentional bias towards rewarding than punishing stimuli observed in people with more optimistic reward expectancies [52–54]. We also expected NegAff mice to take longer to approach (and spend less time with) the light than CompLessNeg mice. It is possible that the handling treatments were not effective at inducing acute relatively positive and negative affective states. However, we chose acute handling manipulations because they have previously been shown to induce differential anxiety-like states in mice [68,69,72, though see 78]. For example, Clarkson et al. [79] found that following tail handling, C57BL/6J mice showed increased anxiety-like behaviours in potentially threatening situations compared with mice that were tunnel handled. In Clarkson et al.’s study (and others) however, the handling method was repeated over several days (10 days in [79]) and in our current study the affective manipulation was only applied once directly before each test. At all other times during training and habituation all mice were handled using their home cage tunnel. Perhaps chronic or repeated manipulations would be necessary to induce affect-related bias in their response towards stimuli in this task. Similarly, in the human literature (on which we based our predictions), the induction of mood states was more prolonged [80] than in our current study, which could account for the dissimilarity between our predicted and observed results.

An alternative explanation for the unexpected influence of affect is that a higher level of arousal, as a result of the tail handling in the NegAff mice, led to enhanced activity (hence shorter obstacle approach speed) compared to CompLessNeg mice who underwent a gentler handling procedure. It is also possible that the NegAff mice were experiencing “relief” from returning to the familiar runway, following isolation in the gravel-bedded box and thus might have been in a more positive affective state during the task than predicted (see [81]). Additionally, if tail handling is aversive, the NegAff mice could have been attempting to flee more quickly from the start-box where they were placed immediately after handling.

Overall, we found significant differences in the behaviour of the two strains of mice during the runway test. On average, DBA/2J mice took longer to complete the runway, approach the obstacle and spent longer within the stimulus presentation area than C57BL/6J mice. Previous studies have found that DBA/2J mice have up to a 30% higher basal metabolic rate and a 0.7°C higher core temperature than C57BL/6Js [82] and that they consume more food in free access tests [83–85], which may explain why they spent longer with the food than C57BL/6Js (because they value food more highly). Moreover Crawley et al.’s (1997) review [64] suggests that C57BL/6J mice typically show more open field locomotion and lower levels of anxiety-like behaviour when compared with DBA/2J mice, even though general strain differences vary between studies [60–64]. Our findings that the DBA/2J mice show longer approach and overall runway latencies could thus also be explained by strain differences in anxiety-like states and/or locomotor tendencies.

Although this task has potential for detecting affect-related variation in response to stimuli in mice, there are several theoretical issues which need to be resolved before it can be developed further. Firstly, the influence of negative affective states on cognition has been more intensively studied than the influence of comparatively more positive affective states, and further research into the interaction between positive affective state and stimulus type in humans is important to advance this field and is relevant to anyone interested in the assessment of either human or animal affect. In addition, significant differences exist between the tasks which have been developed to assess human responses to stimuli and the task we developed here. Human tasks generally use highly focused measures of attention (*e*.*g*. reaction times, gaze direction [86]), whereas few studies have attempted this for animals given the practical difficulties of taking such measures (although see [87–89]). An example of a more focussed behavioural measure which could be used in addition to latency would be investigating and sniffing, however this would require more detailed video footage of mice than we had access to. Differences in results between human and non-human tasks could therefore be attributed to the lack of focussed measures of attention in the mice.

In addition to the theoretical issues, practical refinement of the task would involve using the obstacle [currently used as a marker during the tests for the stimulus presentation area in the runway] from the beginning of the training phase. This would restrict the novelty of the testing situation to the stimuli *per se*, and enable correction for individual differences in motivation not only when analysing the overall latency to reach the home cage (as we did here), but also the measures related to specific areas of the runway, *i*.*e*. the stimulus approach latencies and the time spent in the stimulus presentation area.

To summarise, we have developed a task which successfully differentiated the response of mice to unexpected, task-irrelevant stimuli (*i*.*e*. mice were slower to approach the ‘aversive’ light and spent more time with the ‘attractive’ food), but no clear effect of affect manipulation was observed. Indeed, although we found that following a comparatively more negative affect manipulation, mice tended to be faster to approach the stimuli, particularly the mildly aversive light (and even more so in DBA/2J mice), we found no other effects of affect manipulation. We therefore suggest that theoretical and practical improvements could be made, and conclude that a more prolonged affective state manipulation may be required to dissect the roles of affect and arousal in driving these results. Additionally, we found that the two strains of mice tested behaved differently under test conditions and this should be considered when developing future affective-bias tasks.
